# Excitation/inhibition imbalance in schizophrenia: a meta-analysis of inhibitory and excitatory TMS-EMG paradigms

**DOI:** 10.1038/s41537-024-00476-y

**Published:** 2024-06-15

**Authors:** Orsolya Lányi, Boróka Koleszár, Alexander Schulze Wenning, David Balogh, Marie Anne Engh, András Attila Horváth, Péter Fehérvari, Péter Hegyi, Zsolt Molnár, Zsolt Unoka, Gábor Csukly

**Affiliations:** 1 Centre for Translational Medicine, Semmelweis University, Budapest, Hungary; 2 Department of Psychiatry and Psychotherapy, Semmelweis University, Budapest, Hungary; 3 Neurocognitive Research Center, Nyírő Gyula National Institute of Psychiatry and Addictology, Budapest, Hungary; 4 Department of Biostatistics, University of Veterinary Medicine Budapest, Budapest, Hungary; 5 Institute of Pancreatic Diseases, Semmelweis University, Budapest, Hungary; 6 Institute for Translational Medicine, Medical School, University of Pécs, Pécs, Hungary; 7 Department of Anesthesiology and Intensive Therapy, Semmelweis University, Budapest, Hungary; 8 Department of Anesthesiology and Intensive Therapy, Poznan University of Medical Sciences, Poznan, Poland

**Keywords:** Schizophrenia, Biomarkers

## Abstract

Cortical excitation-inhibition (E/I) imbalance is a potential model for the pathophysiology of schizophrenia. Previous research using transcranial magnetic stimulation (TMS) and electromyography (EMG) has suggested inhibitory deficits in schizophrenia. In this meta-analysis we assessed the reliability and clinical potential of TMS-EMG paradigms in schizophrenia following the methodological recommendations of the PRISMA guideline and the Cochrane Handbook. The search was conducted in three databases in November 2022. Included articles reported Short-Interval Intracortical Inhibition (SICI), Intracortical Facilitation (ICF), Long-Interval Intracortical Inhibition (LICI) and Cortical Silent Period (CSP) in patients with schizophrenia and healthy controls. Meta-analyses were conducted using a random-effects model. Subgroup analysis and meta-regressions were used to assess heterogeneity. Results of 36 studies revealed a robust inhibitory deficit in schizophrenia with a significant decrease in SICI (Cohen’s *d*: 0.62). A trend-level association was found between SICI and antipsychotic medication. Our findings support the E/I imbalance hypothesis in schizophrenia and suggest that SICI may be a potential pathophysiological characteristic of the disorder.

## Introduction

Schizophrenia is a highly complex and severe psychiatric disorder characterized by great diversity in symptom profile, risk factors, treatment response, and prognosis^1^. Schizophrenia has been associated with various genetic and developmental risk factors, molecular and circuit-level impairments, as well as altered neurotransmission, such as striatal dopaminergic dysregulation^2^. However, the exact pathophysiology and the causal association between neural mechanisms and the course of the disorder remain unclear.

Symptoms of schizophrenia are clustered into three main domains of positive, negative, and disorganized/cognitive symptoms, of which the positive dimension responds relatively well to available dopaminergic antipsychotic treatments^2^. However, medication has limited efficacy over the negative and cognitive symptoms, which have been highlighted as core features of disease vulnerability^3^. Therefore, further exploration of the latent neural factors underlying the symptoms is inevitable.

An increasing body of evidence supports the role of γ-amino butyric acid (GABA) and glutamate in the pathophysiology of schizophrenia^4,5^, highlighting a disrupted cortical excitation-inhibition (E/I) imbalance as a neurobiological characteristic of the disorder^6^. E/I imbalance refers to the relative disproportion in the excitatory and inhibitory signaling in the brain, which is necessary for efficient information processing within and between circuits^7^. E/I balance can be studied locally on the synaptic level as well as globally on a network level^8^. Understanding how E/I imbalance defines the symptoms of schizophrenia could open up a new avenue for novel treatment and diagnostic targets^6^.

Disrupted cortical E/I imbalance is supported by postmortem^9^, genetic^10^, and electrophysiological^11^ evidence; furthermore, there is emerging evidence from non-invasive brain stimulation studies using Transcranial Magnetic Stimulation^12^.

Transcranial magnetic stimulation (TMS) is a non-invasive brain stimulation method, which, combined with electromyography (EMG), can be used to assess motor cortical excitability in schizophrenia^13^. When a magnetic pulse (with an intensity reaching the threshold) is applied over the primary motor cortex, a motor-evoked potential (MEP) is elicited in the targeted contralateral peripheral muscle. The amplitude of the MEP can be recorded with EMG. Single- and paired-pulse TMS-EMG paradigms such as Short-Interval Intracortical Inhibition (SICI), Intracortical Facilitation (ICF), Long-Interval Intracortical Inhibition (LICI), and Cortical Silent Period (CSP) evoke excitatory and inhibitory MEP responses, allowing the neurophysiological probe of motor cortical excitability^14^.

Intracortical inhibition can be assessed using paired-pulse paradigms such as SICI and LICI and single-pulse paradigm CSP. SICI is a widely used standard method to elicit an inhibitory response. A subthreshold conditioning stimulus (CS) is followed by a suprathreshold test stimulus (TS) after a 1–4 ms interstimulus interval (ISI), resulting in a relative suppression of the second MEP amplitude^15,16^. Pharmacological studies indicate that SICI is mediated by cortical GABA_A_ receptors, as GABA_A_ agonists such as benzodiazepines enhance SICI^17^.

LICI is a less common inhibitory paired-pulse paradigm, where a suprathreshold CS and TS are delivered with a 100–200 ms ISI^18^. While SICI is mainly GABA_A_ mediated, LICI is argued to be associated with GABA_B_ receptors^19,20^.

CSP is elicited with a single suprathreshold TMS pulse while the individual maintains muscle contraction^21^. After the stimulation, the EMG activity is completely suppressed for a few hundred milliseconds. Although the early phase of CSP is thought to result from spinal inhibition, pharmacological evidence indicates that the later part of the silent period is mediated by GABA_B_ receptors at the cortical level^17^.

Paired-pulse TMS paradigm ICF is a facilitatory response evoked by a similar technique to SICI. A subthreshold CS followed by a suprathreshold TS results in an increase in MEP amplitude when the two stimulations are given with a 10–25 ms ISI^15^. It has been suggested that ICF results from the combined mediation of GABA_A_ and glutamatergic NMDA receptors, but the exact mechanism of intracortical facilitation is still unknown^22,23^.

Schizophrenia has been associated with impaired response in all of the aforementioned TMS-EMG paradigms, with deficits being particularly prominent in the inhibitory paradigms. Nevertheless, the results are inconsistent^24,25^. A previous meta-analysis by Radhu et al.^26^ confirmed a significant difference in SICI between schizophrenia patients and control group, however, the robustness of TMS-EMG paradigms in schizophrenia remains unclear. The aim of this meta-analysis is to fill this gap in the literature and examine the reliability and clinical potential of SICI, ICF, LICI and CSP in schizophrenia by providing a statistical summary of the results published in the literature. Additionally, we hypothesize that finding a disrupted inhibitory response in TMS-EMG paradigms could further support the importance of E/I imbalance as the pathophysiological mechanism behind schizophrenia.

To assess the reliability of TMS-EMG paradigms in schizophrenia we aim to study the association between excitability and antipsychotic medication, symptom severity, and illness duration.

Even though there are other TMS-EMG paradigms of cortical excitability (such as transcallosal inhibition or I-wave facilitation)^27^ here we focus on the four most widely used methods that have been associated with alterations in schizophrenia^28^. The aim of our study is to investigate the reliability of TMS-EMG paradigms in light of the E/I imbalance hypothesis in schizophrenia. Therefore, we did not focus on baseline resting motor threshold (RMT) or motor-evoked potential (MEP) amplitude, but rather on the inhibitory and excitatory responses.

## Methods

The study protocol was predefined and registered on PROSPERO (CRD42022373330) and the meta-analysis was conducted following the recommendations of the Cochrane Handbook and reported according to the PRISMA 2020 guidelines.

### Search and selection

Studies reporting TMS-EMG paradigms (SICI, CSP, ICF, and LICI) in schizophrenia-spectrum populations and healthy control groups were searched for. The systematic search was completed on the 5th of November 2022, using the following search engines: MEDLINE (via PubMed), Embase, and CENTRAL (The Cochrane Central Register of Controlled Trials). The search key used can be found in Supplementary Materials A.

Duplicate removal, title and abstract selection, and full-text selection were performed by two independent review authors (OL + DB and OL + BK, respectively), and disagreements were resolved by a third author (AS). Studies were included if they met the following eligibility criteria: (1) single- or paired-pulse TMS paradigms were applied in the primary motor cortex and the response was measured with EMG, (2) means and standard deviations for SICI, CSP, ICF, or LICI were reported for both patient and control groups, (3) all patients in the schizophrenia group met the DSM-IV or DSM-5 criteria for schizophrenia, schizoaffective disorder or first episode psychosis, (4) a detailed methodological description was provided with the calculation of the TMS-EMG outcome measures. Review articles, case reports, conference abstracts, and non-English language publications were excluded, as well as studies measuring TMS excitation with EEG.

### Data synthesis

From the eligible articles, means and standard deviations of SICI, CSP, ICF, LICI, and resting motor threshold (RMT) were extracted, as well as group-level demographic data (sample size, age, sex) for both study populations. Diagnosis, PANSS scores, medication status (medicated/unmedicated), type of antipsychotic medication (atypical/typical or combination), CPZ-equivalent daily medication dose, and illness duration were extracted for the patient group. Most articles used a combined inclusion criteria for schizophrenia and schizoaffective disorder diagnoses therefore our pooled diagnostic categories are overlapping (Table 1). Interstimulus interval (ISI), stimulation intensity, type of TMS stimulator and coil, stimulation side, target muscle, and type of EMG electrode were collected for the TMS-EMG paradigms. Where available, data were also extracted separately for medicated and unmedicated patient groups. Where multiple ISI or intensity variations were available, or results for different medication groups were reported separately, total weighted averages were calculated for each article. Mean length of CSP was collected in milliseconds. The calculation of outcome measures for paired pulses varied across articles. SICI was reported, for example, as a ratio of [conditioned/unconditioned MEP] expressed raw number or percentage, as the reciprocal [unconditioned/conditioned MEP] or as the percentage of inhibition [1- conditioned/unconditioned MEP]. In this meta-analysis, all paired-pulse outcome measures are calculated as [conditioned/unconditioned MEP × 100] where SICI and LICI are ratios smaller than 100 whereas ICF is a ratio greater than 100. In our calculation, any number below 100 represents an inhibition, and smaller numbers represent increased inhibition. Numbers greater than 100 represent excitatory responses. Where results were reported differently, means were adjusted to match our calculation. WebPlotDigitizer^29^ was used for graphical data extraction. Data extraction was performed by OL and BK independently and disagreements were resolved by AS.

**Table 1:** Basic characteristics of included studies.

Reference	*N* SCH (female)	*N* control (female)	Mean age SCH (SD)	Mean age control (SD)	Diagnosis	TMS-EMG paradigms	Stimulation intensity	TMS device	Position of EMG electrode
Ahlgrén-Rimpiläinen et al.^40^	11 (5)	9 (5)	42.6 (13.7)	36.1 (8.4)	SCH*	CSP	60–80% of maximum stimulator intensity	Cadwell MES-10. round coil	Dominant ADM
Bagewadi et al.^41^	39 (15)	28 (13)	28.6 (4.5)	26.5 (4.3)	SCH*	SICI. ICF	CS: 80% RMT. TS: stimulation intensity to elicit 1 mV MEP. ISI: 3 ms; 10 ms	MagPro R30	Right FDI
Bajbouj et al.^42^	16 (4)	16 (4)	31.3 (10.5)	33.6 (10.2)	SCH**	CSP	80% of maximum stimulator intensity	2 x Magstim 200. figure-of-eight coil	Right FDI
Bridgman et al.^43^	11 (4)	13 (3)	38.5 (9)	35.5 (10.5)	SCH + SAD*	SICI. CSP	SICI: CS: 80% RMT. TS: stimulation intensity to elicit 1 mV MEP. ISI: 2-4 ms CSP: 140% of RMT	2 x Magstim 200. figure-of-eight coil	Right APB
Carment et al.^44^	25 (7)	25 (7)	31 (9)	30 (7)	SCH*	SICI	CS: 80% RMT. TS: 120% RMT. ISI: 2 ms	2 x Magstim 200. figure-of-eight coil	Right FDI
Daskalakis et al.^45^	30 (12)	15 (5)	32.7 (9.1)	28.4 (8.6)	SCH**	SICI. ICF. CSP	SICI. ICF: CS: 80% RMT. TS: stimulation intensity to elicit 0.5–1.5 mV MEP. ISI: 2. 4. 10. 15. 20 ms CSP: 140% of AMT	2 x Magstim 200. figure-of-eight coil	Both FDI
Daskalakis et al.^46^	16 (5)	10 (3)	31.2 (8)	32.8 (7.4)	SCH**	SICI. ICF. CSP	SICI. ICF: CS: 80%. TS: stimulation intensity to elicit 0.5–1.5 mV MEP. ISI: 2. 4. 10. 15. 20 ms CSP: 140% of AMT	2 x Magstim 200. figure-of-eight coil	Right APB
Du et al. ^47^	26 (7)	36 (17)	36.8 (13.4)	41.1 (14.1)	SCH	SICI. ICF	CS: 80% RMT. TS: 120% RMT. ISI: 1–3; 9–21 ms	Magstim 200. figure-of-eight coil	Right FDI
Du et al. ^49^	35 (7)	29 (13)	36.9 (13.7)	41.1 (14.2)	SCH	SICI. ICF	CS: 80% RMT. TS: 120% RMT. ISI: 1–3; 9–21 ms	Magstim 200. figure-of-eight coil	Right FDI
Du et al. ^48^	24 (7)	30 (14)	36.5 (13.5)	42.2 (13.6)	SCH + SAD	SICI	CS: 80% RMT. TS: 120% RMT. ISI: 1–3 ms	Magstim 200. figure-of-eight coil	Right FDI
Eichammer et al.^50^	21 (11)	21 (11)	29 (8.2)	31.3 (9.6)	FEP***	SICI. ICF	CS: 5% of max. stimulator intensity. TS: stimulation intensity to elicit 0.5–1 mV MEP. ISI: 1–30 ms	Magstim 200. figure-of-eight coil	Right APB
Fitzgerald et al.^74^	40 (8)	22 (7)	28.2 (9.2)	31.8 (8.9)	SCH + SAD*	SICI. ICF. CSP	CS: 5% below AMT. TS: stimulation intensity to elicit 0.5–1 mV MEP. ISI: 1. 2. 3. 4. 10. 15. 20 ms CSP: 140% of RMT	Magstim 200. figure-of-eight coil	Right APB
Fitzgerald et al.^53^	22 (5)	21 (7)	28.8 (7.9)	32.2 (8.9)	SCH*	SICI. ICF	CS: 5% below AMT. TS: stimulation intensity to elicit 0.5–1 mV MEP. ISI: 1. 2. 3. 4. 10. 15. 20 ms CSP:10. 20. 30. 40% above RMT	2 x Magstim 200. figure-of-eight coil	Right APB
Fitzgerald et al.^52^	18 (6)	8 (2)	30.7 (7.1)	29.1 (6)	SCH**	LICI	CS and TS: intensity to elicit 0.5–1.0 mV MEP. ISI: 100 ms	2 x Magstim 200. figure-of-eight coil	Right APB
Fitzgerald et al.^51^	26 (8)	18 (3)	32.4 (8.5)	31 (5.5)	SCH**	SICI. ICF. CSP	CS: 5% below AMT. TS: stimulation intensity to elicit 0.5–1 mV MEP. ISI: 2. 15 ms CSP: 120% of RMT	2 x Magstim 200. figure-of-eight coil	Right APB
Frank et al.^54^	24 (9)	24 (9)	29 (8.7)	29 (8.4)	FEP**	CSP	150% of RMT	2 x Magstim 200. figure-of-eight coil	Right ADM
Goodman et al.^55^	23 (4)	23 (3)	33.8 (8.7)	33.3 (9.15)	SCH*	SICI. LICI. ICF. CSP	SICI. ICF: CS: 80% RMT. TS: stimulation intensity to elicit 0.5–1 mV MEP. ISI: 2. 4. 10. 15. 20 ms LICI: CS and TS intensity to elicit 1 mV MEP. ISI: 100 ms CSP: 140% of RMT	Magstim 200. figure-of-eight coil	Right APB
Hare et al.^56^	23 (7)	29 (14)	37 (13.5)	33.3 (9.2)	SSD	SICI	CS: 80%. TS: 120% of RMT. ISI: 3 ms	Magstim 200. figure-of-eight coil	Right FDI
Hasan et al.^59^	21 (8)	21 (8)	33.2 (8.4)	31.52 (7.6)	SCH	CSP	NA	MagPro-X100. figure-of-eight coil	Both FDI
Hasan et al.^60^	18 (4)	18 (4)	25.3 (6.3)	24.4 (2.4)	FEP*	SICI. ICF. CSP	CS: 80% RMT. TS: stimulation intensity to elicit 0.5–1.5 mV MEP. ISI: 3. 15 ms CSP: 120% of RMT	MagPro-X100. figure-of-eight coil	Right FDI
Hasan et al.^57^	25 (7)	41 (21)	29.9 (8.5)	24.4 (2.4)	FEP	SICI. ICF. CSP	CS: 80% RMT. TS: stimulation intensity to elicit 0.5–1.5 mV MEP. ISI: 3. 15 ms CSP: 120% of RMT	MagPro-X100. figure-of-eight coil	Both FDI
Hasan et al.^58^	9 (3)	9(3)	34 (10.2)	28.9 (12.5)	SCH*	SICI. CSP	CS: 80% RMT. TS: stimulation intensity to elicit 0.5–1.5 mV MEP. ISI: 3 ms CSP: 120% of RMT	MagPro-X100. figure-of-eight coil	Both FDI
Kaster et al.^61^	16 (5)	14 (4)	33.3 (10.9)	34.9 (10.7)	SCH*	SICI. ICF. CSP	NA	2 x Magstim 200. figure-of-eight coil	Right APB
Lindberg et al.^62^	28 (4)	31 (15)	32 (6.7)	30.3 (7.7)	SCH + SAD	SICI. CSP	CS: 90% of AMT. TS: intensity to elicit a MEP of 1.5 mV. ISI: ? CSP: ?	Figure-of-eight coil	Right FDI
Liu et al.^63^	26 (8)	38 (10)	33.2 (10.2)	34.3 (10.2)	SCH + SAD**	SICI. ICF. CSP	CS: 80% of RMT. TS: suprathreshold. ISI: 3 ms CSP: 140% of RMT	Magstim 200. figure-of-eight coil	Right APB
Mehta et al.^64^	54 (27)	45 (22)	31.4 (8)	30.68 (6.6)	SCH**	SICI. LICI	SICI: CS 80% RMT. TS: intensity to elicit 1 mV MEP. ISI: 3 ms LICI: CS and TS: intensity to elicit 1 mV MEP. ISI: 100 ms	MagPro R30. figure-of-eight coil	Right FDI
Mehta et al.^65^	76 (39)	125 (49)	32.4 (8.7)	30.7 (7.5)	SCH	SICI. LICI. CSP	SICI: CS 80% RMT. TS: intensity to elicit 1 mV MEP. ISI: 3 ms LICI: CS and TS: intensity to elicit 1 mV MEP. ISI: 100 ms CSP: suprathreshold intensity to elicit 1 mV MEP	MagPro R30. figure-of-eight coil	Right FDI
Miyazawa et al.^66^	12 (5)	27 (16)	39.1 (9.5)	37.4 (13.1)	SCH + SAD**	CSP	120% RMT	Magstim Rapid 2	Right FDI
Oxley et al.^67^	12 (4)	12 (4)	34 (6.9)	37.4 (13.1)	SCH	SICI	CS: 80% RMT. TS: stimulation intensity to elicit 0.5–1 mV MEP. ISI: 2	2 x Magstim 200. figure-of-eight coil	Right APB
Pascual et al.^68^	15 (2)	7 (1)	43.7 (10.6)	40.6 (15.7)	SCH**	SICI. ICF	CS: 80% RMT. TS: approximately 130% of RMT. ISI: 2. 12 ms	2 x Magstim 200. figure-of-eight coil	Both FDI
Roeh et al.^69^	17 (3)	16 (5)	36.8 (12.1)	37.3(11.8)	SCH	SICI. ICF. CSP	CS 80% RMT. TS: intensity to elicit 1 mV MEP. ISI: 3. 15 ms CSP: 150% RMT	MagPro-X100. figure-of-eight coil	Both FDI
Schecklmann et al.^70^	81 (34)	49 (30)	32.8 (10.9)	32.4 (9.3)	SCH**	SICI. ICF. CSP	CS: 90% of AMT. TS: intensity to elicit a MEP of 1 mV. ISI: 2. 10 ms CSP: NA	2 x Magstim 200. figure-of-eight coil	Right ADM
Soubasi et al.^71^	51 (18)	51 (18)	34.4 (8.5)	34.4 (8.5)	SCH	CSP	130% RMT	Magstim 200. round coil	Both APB
Takahashi et al.^90^	20 (11)	20 (10)	27.4 (6.5)	27.8 (3.5)	FEP	SICI. ICF	CS: 80% RMT. TS: 130% RMT. ISI: 3. 10 ms	2 x Magstim 200. figure-of-eight coil	Right FDI
Tang et al.^72^	17 (8)	28 (13)	31.71 (9)	20.93 (6.2)	SCH*	SICI. ICF. CSP	CS 80% RMT. TS: intensity to elicit 1 mV MEP. ISI: 3. 10 ms CSP: 120% RMT	MagPro-X100. figure-of-eight coil	Right APB
Wobrock et al.^73^	29 (8)	44 (23)	29.8 (8.5)	33.9 (9.1)	FEP*	SICI. ICF. CSP	CS: 80% of AMT. TS: intensity to elicit a MEP of 1.5 mV. ISI: 3. 7 ms CSP: 120% RMT	MagPro-X100. figure-of-eight coil	Both FDI

Asterisks: asterisks in the diagnosis column indicate medication status. No asterisk: mixed patient population where some patients are medicated and some are unmedicated, or information on medication status is unavailable; *: all patients are medicated, **: data for both medicated and unmedicated patients are available, ***: all patients are unmedicated.

*CSP* cortical silent period, *SICI* short-interval intracortical inhibition, *ICF* intracortical facilitation, *LICI* long-interval intracortical inhibition, *ISI* interstimulus interval, *MEP* motor-evoked potential, *RMT* resting motor threshold, *AMT* active motor threshold, *CS* conditioning stimulus, *TS* test stimulus, *ADM* abductor digiti minimi, *FDI* first dorsal interosseus, *APB* abductor pollicis brevis, *SCH* schizophrenia, *SAD* schizoaffective disorder, *FEP* first episode psychosis, *SSD* schizophrenia-spectrum disorder.

### Statistical analysis

Data were analyzed with R (version 4.0.3)^30^ in RStudio^31^, using the meta^32^, metafor^33^, and dmetar^34^ packages. A random-effects model was applied to estimate the pooled mean difference with 95% confidence intervals. We anticipated variations in the true effect size across studies. To account for sampling error and such variances resulting from differences in experimental conditions, demographics and the heterogeneity of the patient population, we used a random-effects model. Between study variance of the true effect size was estimated with the Tau^2^ using the restricted maximum likelihood method (REML)^35^. The Hartung-Knapp adjustments^36^ were applied for the calculation of the confidence interval around the pooled effect size to compensate for small effect sizes reported in the articles. We used the I^2^ test to evaluate the amount of heterogeneity due to variability other than sampling error^37^. Forest plots were used to visualize mean differences and pooled effect sizes, and funnel plots were used to present publication bias.

In order to understand potential sources of heterogeneity, we first conducted exploratory analyses and assessed the distribution of data and evaluated the linear relationships between moderator variables such as demographics, disorder characteristics, experimental design and measurement details. When exploratory analyses revealed tendency-like associations (for example raw mean differences between medicated and unmedicated patients) or linear relationships (for example SICI of schizophrenia group and illness duration), we included all moderator variables in meta-regressions (metareg R function) and subgroup analyses.

### Study risk of bias (ROB) assessment

Following the recommendations of the previous Cochrane Handbook on case-control studies, the Newcastle-Ottawa Scale (NOS)^38^ was used for risk of bias assessment. The checklist for TMS studies by Chipchase et al.^39^ was used to account for potential bias in the TMS methodology. The NOS was modified to include an additional point based on the outcome of the TMS score. Articles scoring above 70% on the TMS checklist were assigned an extra point in the NOS assessment, while those below 70% did not receive this adjustment. With the additional TMS score in the NOS, the scale ranged from 0–10. Without any published scoring guidelines, we used the following cutoff scores to determine ROB: 0–4 high risk, 5–7 moderate risk, and 8–10 low risk. ROB assessment was completed independently by two authors (OL and BK).

## Results

### Search and baseline characteristics

A total of 1715 case-control articles were identified by our search key and 80 reports were assessed for full-text selection. A final sample of 36 studies^40–50,51–74^ met the eligibility criteria and were included in the meta-analysis (flowchart of selection and reasons for exclusion in Supplementary Materials B).

The baseline characteristics of the included studies are presented in Table 1. Twenty-eight studies included SICI with 794 schizophrenia patients and 817 controls (mean age SCH = 32.25, min = 25.30, max = 38.5; mean age HC = 32.25, min = 20.93, max = 40.22). Eighteen studies reported SICI for medicated patients separately and 8 for unmedicated patients. Schizophrenia patients had a mean illness duration of 6.80 years (min = 0 in FEP, max = 16.40), a mean of 69.71 total PANSS score (min = 50.50, max = 92.40), and medicated patients were treated with a daily mean of 425.54 mg chlorpromazine (CPZ) equivalent antipsychotic dose (min = 283.97, max = 860.80).

Our second outcome measure, CSP was reported in 23 articles (total N control = 662, schizophrenia = 623), of which 17 reported data of medicated patients and 7 of unmedicated patients. The mean age of schizophrenia patients was 33.35 (min = 25.30, max = 42.60), and 31.81 for the control group (min = 20.93, max = 37.41). The patient population was characterized by an average of 7.07 years of illness duration (min = 0 in FEP, max = 18.30), a mean PANSS total of 71.76 (min = 50.50, max = 101) and a mean of 425.89 mg daily CPZ equivalent medication dose (min = 266.31, max = 860.80).

Twenty articles reported on ICF and only four articles reported on LICI, for descriptive statistics see the Supplementary Materials C.

### Short-interval intracortical inhibition (SICI)

**Fig. 1:**
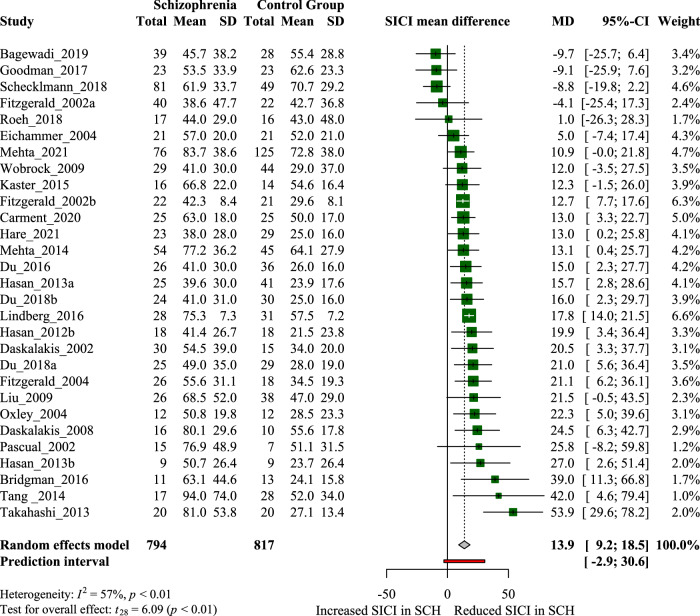
Forest plot of short-interval intracortical inhibition (SICI) shows the mean difference of SICI between schizophrenia (SCH) and healthy control groups (HC). SICI is expressed as [conditioned/unconditioned MEP × 100]. Articles from zero to left indictate an increased inhibition in SCH whereas articles from zero to right indicate a decreased inhibition in SCH.

Results from the random-effects model revealed a robust (Cohen’s *d*: 0.62) and significant reduction in SICI in schizophrenia patients compared to healthy controls (Fig. 1). The pooled mean difference shows that the inhibitory response is 13.85% (95% CI: 9.19–18.51) smaller in schizophrenia. The results remained robust after sensitivity analysis (total mean difference after removing moderate to high ROB articles: 13.36%, 95% CI: 7.99–18.04). Between-study heterogeneity is moderate (*I*^2^ = 57%, *p* < 0.001), a part of which can be explained by moderate to high ROB (heterogeneity of the sensitivity analysis: *I*^2^ = 50%, *p* = 0.01). In addition to ROB^75^, experimental conditions such as the evaluation of task-related SICI^41^ and cannabis use^55^ could account for cases where SICI was found to be higher in schizophrenia.

**Fig. 2:**
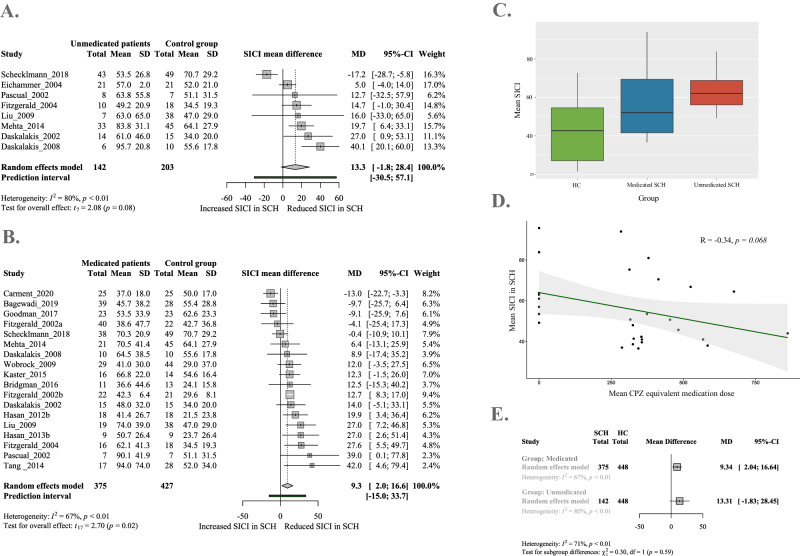
Antipsychotic medication and short-interval intracortical inhibition (SICI) in schizophrenia. **A** Forest plot for SICI between unmedicated schizophrenia patients (SCH) and healthy control group (HC). **B** Forest plot for SICI between medicated SCH patients and HC. **C**, **D** Exploratory analysis of medication status and mean CPZ equivalent medication dose of SICI. **E** Summary of total effect sizes for subgroup analysis of SICI between medicated and unmedicated SCH and HC.

Multiple subgroup analyses were conducted to further elucidate potential sources of heterogeneity and investigate the association between SICI and medication status. Exploratory analyses revealed a tendency-like association between SICI and medication status as well as SICI and CPZ equivalent antipsychotic dose (see Fig. 2D). Based on further exploratory analysis, the effect of medication status seems to be more defined than the effect of medication dose (Supplementary Materials D). However, subgroup analysis did not confirm that there is a difference in the extent to which medicated (*N* = 375) and unmedicated (*N* = 142) schizophrenia patients differ from the healthy population (Fig. 2E). Meta-regressions conducted with covariates such as symptom severity (PANSS total, positive, negative and general scores), illness duration, age and sex did not reveal a significant effect on the pooled effect size (Supplementary Material D). Exploratory analysis revealed a tendency-like association between the proportion of females and SICI, suggesting that inhibitory response was greater in samples with more males than females.

Additionally, subgroup analysis did not show any variance of SICI across different TMS conditions (stimulation side, device, target muscle, interstimulus interval), diagnoses or date of publication.

### Cortical silent period (CSP)

**Fig. 3:**
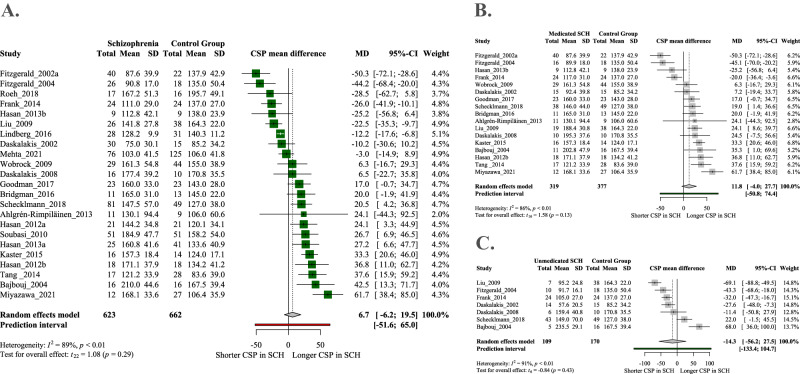
Forest plots of cortical silent period (CSP) in schizophrenia. **A** Results of the random-effect meta-analysis showing the mean difference of CSP between schizophrenia patients (SCH) and healthy control groups (HC). CSP is measured in milliseconds, length of CSP is proportional to the extent of inhibition. Articles from 0 to left found shorter CSP in SCH whereas articles from 0 to right reported longer CSP in SCH. **B** Forest plot of CSP between unmedicated SCH patients and H. **C** Forest plot of CSP between medicated SCH patients and HC.

Results for CSP did not show any difference between schizophrenia patients and control group (Fig. 3). The pooled effect size showed high heterogeneity between studies (*I*^2^ = 89%, *p* < 0.001) and a wide confidence interval around the pooled total effect size (6.69, CI: −6.16–19.54). Furthermore, the large inconsistency between published results is reflected in the range of CSP within the control group (83.6–195.7 ms), which could be the result of biological variability or measurement bias.

Subgroup analyses and meta-regressions of experimental conditions, demographic variables, and medical variables failed to identify any single moderator variable that could account for the variance found between studies. Nevertheless, a tendency-like association was found between medication status and CSP. Unmedicated patients exhibited a slightly shorter CSP (reduced inhibition), whereas medicated patients showed a slightly longer CSP compared to the control group (Fig. 3). However, these results are not significant and are highly heterogeneous.

### Intracortical facilitation (ICF), long-interval intracortical inhibition (LICI), and resting motor threshold (RMT)

**Fig. 4:**
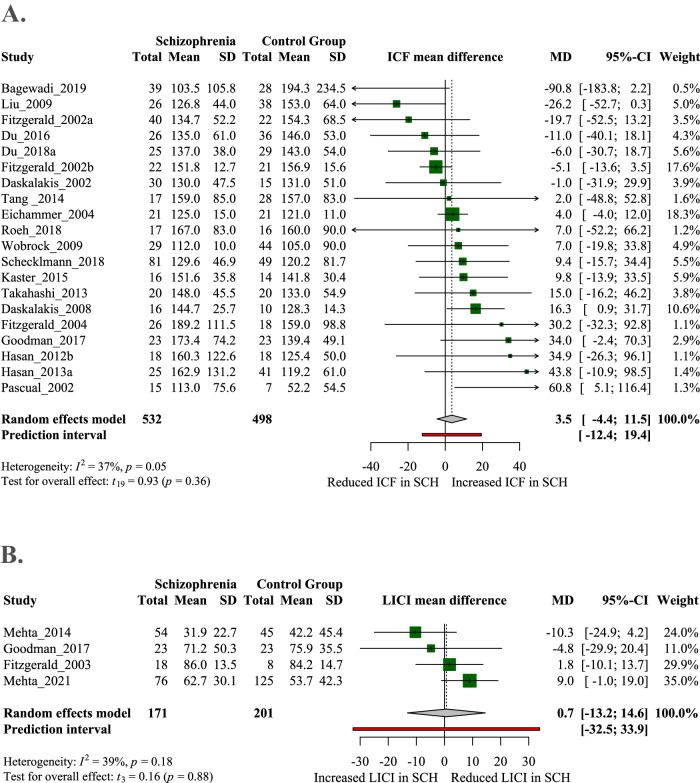
Intracortical facilitation (ICF) and long-interval intracortical inhibition (LICI) in schizophrenia. **A** Forest plot of ICF. Articles to the left of zero indicate decreased ICF in schizophrenia (SCH) while those to the right show increased ICF in SCH. No significant difference was found between the two groups. **B** Forest plot of LICI. Articles from zero to left reported increased inhibition in SCH whereas articles from zero to right found decreased inhibition in SCH. No significant difference was observed between the two groups, and a conclusion cannot be drawn from these results due to the small number of included articles.

We found no clear difference between schizophrenia patients and control groups in terms of excitatory response ICF or the inhibitory response LICI (Fig. 4). Results of ICF are reliable, the relatively big sample size (SCH = 532, HC = 498) and the low between-study heterogeneity (*I*^2^ = 37%, *p* = 0.005) confirm that there is no clear difference between the two groups. Sensitivity analysis (excluding moderate to high ROB articles) did not improve or change the overall results of ICF (total mean difference = 0.45, 95% CI: −12.66–13.56). Subgroup analyses and meta-regressions (depending on the class of variables) did not account for heterogeneity in experimental conditions, medical conditions, demographic variables, or time of publication.

Due to the small number of articles included (*N* = 4) and the relatively small pooled sample size (SCH = 170, HC = 201), the results of LICI are not sufficient to draw conclusions from.

A powerful analysis of RMT (Supplementary Materials E), which included 33 studies and an impressive sample size (SCH = 902, HC = 932), did not reveal any group differences in baseline excitability. A sensitivity analysis based on ROB did not change the overall effect (total mean difference = 1.69, 95% CI: −0.37–3.75).

### Risk of bias, publication bias, and heterogeneity

Of the 36 articles included, 2 articles had a combined high risk of bias on the NOS and the TMS checklists, 13 had moderate risk and 21 articles received a low risk of bias score (results available online at https://osf.io/8cef3/). Funnel plots and Egger’s test were used to assess publication bias across the included studies. The funnel plots (Supplementary Materials F) revealed relative symmetry for all TMS-EMG paradigms except CSP, which raised some concerns about potential publication bias. No significant publication bias was detected with the Egger’s test (Supplementary Materials G).

## Discussion

In this meta-analysis, we statistically pool data from 36 articles investigating cortical excitability with single- and paired-pulse TMS-EMG paradigms. The aim of our research was to find evidence for the disrupted E/I balance in schizophrenia and to determine the reliability and predictive value of TMS-EMG paradigms in schizophrenia.

To our knowledge, this is the first meta-analysis to statistically confirm the widely reported reduction in SICI in medicated and unmedicated schizophrenia patients. Our results confirmed the hypothesized difference between schizophrenia patients and healthy controls with a relatively large pooled sample (SCH = 794, HC = 817) and moderate to large effect size (Cohen’s *d* = 0.62). The pooled mean difference shows a 13.85% (95% CI = 9.19–18.51) between-group difference in the inhibitory response (Fig. 1), suggesting that intracortical inhibitory deficit in schizophrenia is indeed clinically relevant. Results for SICI remained unchanged after sensitivity analysis, confirming that the effect is stable. Furthermore, we only found three articles that contradicted our results^41,55,70^, and there are potential methodological explanations in all three cases (task-related SICI as opposed to resting state, cannabis use, and high risk of bias).

Although previous research suggested that SICI could be associated with antipsychotic medication^45^ and symptom severity^46^, our study was underpowered to confirm these results with subgroup analysis or meta-regression (Supplementary Material D). Nevertheless, exploratory analysis revealed a trend-level association between SICI and medication status as well as medication dose, suggesting that unmedicated patients might exhibit reduced SICI. Based on our exploratory analyses on medication dose, we propose that medication status itself rather than antipsychotic dose could be driving the effect. However, further research is needed to better understand these associations and how the different antipsychotic agents affect SICI.

There are several possible explanations for these ambiguous results on the effect of antipsychotics. First, we had a relatively small number of studies including unmedicated schizophrenia patients (*N* = 8), with a small pooled sample size (*N* = 142). Second, the patient population of the studies including unmedicated patients was small (*N* of patients: mean = 17.75, range: 6–43), and inclusion criteria varied across studies. Some studies included antipsychotic-naive patients^50^, whereas others included patients who had not been receiving antipsychotic treatment for the past 3 months^51^. The patient populations also differed in whether they received benzodiazepines, which has been confirmed to increase SICI^24^. Regrettably, data on benzodiazepine intake was not reported in the included articles therefore we were unable to control for its confounding effect. Lastly, medicated patients were treated with various types and combinations of antipsychotics with different receptor binding profiles^76^. We were only able to extract data and assess the moderator effect of clozapine directly, however, due to the small number of articles (*N* = 3) and the small sample size (*N* = 58), no conclusions could be drawn on its effect. Due to these limitations, our study lacked the statistical power to provide significant evidence on the impact of antipsychotic medication on SICI. Nevertheless, the notable correlation observed at a trend level between medication status, medication dose and SICI (Fig. 2C, D) suggests that antipsychotic medication could play an important role in reducing the magnitude of the SICI inhibitory deficit. This question should be targeted in future research to confirm the effect of medication on SICI.

The reliability of SICI is also supported by the fact that diversity in TMS conditions and experimental designs did not change the overall effect of SICI. Articles included in the present study applied a variety of experimental designs and TMS conditions (Table 1), with significant differences in stimulation intensity, used device, coil type, target muscle, direction of induced current, or pulse shape. The technical specifications within the TMS protocol may impact the evoked response^75,77^, nevertheless, adjusting for variations in experimental setup through subgroup analysis and meta-regression did not alter the overall effect.

Our results of SICI are in line with the previous meta-analysis published in 2013 by Radhu et al.^26^, further supporting the reliability of SICI in schizophrenia.

In contrast to SICI, our results showed no clear difference in CSP, ICF, or LICI between schizophrenia patients and healthy controls. This implies that the dysfunction in motor cortical excitability in schizophrenia may be specific to GABA_A_ receptors involved in SICI^17^.

## Limitations and heterogeneity

This study has several limitations that should be considered. Firstly, the number of studies included in the LICI analysis was too small, completely limiting the conclusions that can be drawn from this analysis. Secondly, there is high heterogeneity in the case of ICF and CSP, which has to be considered when interpreting the findings.

The high degree of heterogeneity found in CSP is in line with the meta-analysis by Miyazawa et al.^78^. We hypothesize that this heterogeneity might be due to several factors, including differences in TMS-EMG protocols (e.g., intensity, muscle contraction, pulse shape, determining the end of CSP)^14^, biological variability^79^, and the reporting of results with small sample sizes (SCH range: 9–81, HC range: 9–125) without accounting for the skewness of the data. Consequently, we suggest that future research should take such methodological considerations into account and report the distribution of the data when using the mean as an outcome.

There is considerably less heterogeneity underlying ICF. Our study was underpowered to account for this heterogeneity by testing the effect of clinical and methodological confounding factors.

The high heterogeneity observed in ICF and CSP may be due to confounding factors that we were not able to control for due to the small number of studies included when conducting a subgroup-analysis or meta-regression.

Therefore, the potential impact of clinical and methodological confounding variables should be further explored in future studies for all TMS-EMG paradigms. Larger samples are needed and the technical factors of TMS-EMG paradigms (such as stimulation intensity, pulse shape, ISI, direction of induced current, etc.) should be evaluated with more methodological rigor.

Moreover, there is a need for additional research on the impact of clinical variables (including diagnosis, medication type and dosage, and symptom severity) and demographic variables (such as age and sex) that were beyond our study’s capacity to thoroughly investigate.

## Clinical and methodological implications

The results of the present meta-analysis have several clinical and methodological implications. Our results of SICI are in line with the gabaergic hypothesis of schizophrenia, and contribute to the growing evidence supporting the E/I imbalance in schizophrenia^80,81^. The E/I hypothesis is a promising model to link the neurodevelopmental and the dopaminergic hypotheses of schizophrenia. Understanding the role of E/I imbalance in the pathomechanism of schizophrenia could offer novel approaches for developing treatment targets within the GABA system to improve the cognitive and negative symptom domains of schizophrenia^6^.

Our results confirm that SICI is a reliable and robust TMS-EMG protocol showing a clear inhibitory deficit in schizophrenia regardless of experimental conditions and demographic variables. Therefore, our results raise the possibility that SICI, in combination with other reliable markers, may contribute to supporting current clinical diagnosis with physiological data. The diagnostic utility of TMS-EMG paradigms in the field of neurology is well established^82^, therefore we believe the results of this meta-analysis provide a foundation for future clinical research and confirm that the inhibitory deficits of SICI are robust enough to have a clinical potential.

The scope of this study limited us to assess the specificity and sensitivity of SICI in schizophrenia, therefore we argue that future research should further investigate the potential moderating factors of SICI in order to validate its clinical relevance and evaluate its diagnostic properties. Previous research comparing other psychiatric disorders such as OCD or depression to schizophrenia have found different patterns of excitability deficits in each disorder^26,65^, however, the specificity of SICI in schizophrenia have not been studied yet.

SICI might also have the potential to be investigated as a target for response prediction of pharmacological agents or neuromodulation. Non-invasive therapeutic brain stimulation techniques such as repetitive TMS (rTMS) or transcranial direct current stimulation (tDCS) have been shown to change the excitability of the stimulated circuits^83^, and studies confirm this by showing alterations in TMS-EMG paradigms in response to rTMS or tDCS^51,54,58,61^. In addition to response prediction, SICI may be further investigated as a measure of individual brain state to support the timing of state-dependent or closed-loop brain stimulation^84,85^.

Here we suggest that future studies should focus on investigating the diagnostic and prognostic value of SICI in larger patient samples and explore its potential use as a supportive biomarker or a marker for therapeutic response prediction. Future research should also further investigate the role of antipsychotic medication and its potential impact on SICI in schizophrenia.

Our results of CSP, ICF, and LICI have limited clinical relevance due to the high degree of heterogeneity and the smaller number of included studies.

In order to reduce heterogeneity and confounding due to methodological differences in the TMS-EMG paradigms, we suggest that future clinical research should consider some methodological aspects. First, we encourage the use of quality checklists (such as the one by Chipchase et al.^39^) to promote standardized reporting of results. In addition, to avoid misinterpretation, we believe that it is also of great importance to be consistent with the language used when referring to paired-pulse paradigms and to clearly state whether SICI or LICI refers to the amount of inhibition or to the ratio number calculated from the conditioned and unconditioned MEP amplitude.

TMS-EMG is a well-established tool to probe cortical excitability which fits well with other electrophysiological methods that have been suggested to be a marker of E/I imbalance^12^. For a better understanding of the E/I imbalance and the clinical potential of SICI, it would be worthwhile in the future to compare TMS-EMG paradigms to other electrophysiological methods such as EEG gamma-band power^86^, spectral slope^87^, event-related potentials^88^ or TMS-EEG^89^.

## Conclusion

This meta-analysis provides evidence for a significant decrease in SICI in schizophrenia patients, supporting the hypothesis that gabaergic inhibitory dysfunctions play an important role in the pathophysiology of the disorder. Here we argue that the E/I imbalance is a robust physiological characteristic of the disorder which can be assessed with SICI. Our results suggest that SICI is a promising candidate as one of the potential physiological characteristics that could complement the observational diagnosis of schizophrenia with physiological data or serve as a marker for therapeutic response prediction.

### Supplementary information

Supplementary Materials

## Data Availability

We have made all data for this meta-analysis publicly available. The data extraction table, the results of the risk of bias assessment and the R code for the statistical analysis can be found online in the OSF repository: https://osf.io/8cef3/.
