# Supplementary material for: Excitation/inhibition imbalance in schizophrenia: a meta-analysis of inhibitory and excitatory TMS-EMG paradigms
Source: Schizophrenia (Heidelb). 2024 Jun 15;10(1):56. doi: 10.1038/s41537-024-00476-y (PMC11180212; doi:10.1038/s41537-024-00476-y)
Supplement: Supplementary file 1 — Supplementary Materials [file 41537_2024_476_MOESM1_ESM.doc]

# SUPPLEMENTARY MATERIALS

Excitation/Inhibition Imbalance in Schizophrenia: A Meta-Analysis of Inhibitory and Excitatory TMS-EMG Paradigms

# Orsolya Lányi^1,2^, Boróka Koleszár^2^, Alexander Schulze Wenning, MD^1^, David Balogh, MD^2^, Marie Anne Engh, MD^1^, András Horváth, MD, PhD^3^, Péter Fehérvari, PhD^4,1^, Péter Hegyi, MD, PhD^1,5,6^, Zsolt Molnár, MD, PhD^7,1,8^, Zsolt Unoka MD, PhD^2^, Gábor Csukly, MD, PhD^2*^

# Centre for Translational Medicine, Semmelweis University, Budapest, Hungary

# Department of Psychiatry and Psychotherapy, Semmelweis University, Budapest, Hungary

# National Institute of Mental Health, Neurology and Neurosurgery, Neurocognitive Research Center, Budapest, Hungary

# Department of Biostatistics, University of Veterinary Medicine Budapest, Budapest, Hungary

# Institute of Pancreatic Diseases, Semmelweis University, Budapest, Hungary

# Institute for Translational Medicine, Medical School, University of Pécs, Pécs, Hungary

# Department of Anesthesiology and Intensive Therapy, Semmelweis University, Budapest, Hungary

# Department of Anesthesiology and Intensive Therapy, Poznan University of Medical Sciences, Poznan, Poland

*Corresponding author:

Gábor Csukly, MD, PhD
Semmelweis University, Department of Psychiatry and Psychotherapy
Budapest, Hungary, Balassa u. 6, 1083
Email: csukly.gabor@med.semmelweis-univ.hu
Telephone: +36208250174

**A) Search key**

The following search key was used for our meta-analysis:

(schizophrenia OR schizoaffective OR psychotic OR psychosis OR schizophrenic) AND ('TMS EMG' OR 'motor evoked potential' OR 'TMS MEP' OR 'short-interval intracortical inhibition' OR SICI OR 'long-interval intracortical facilitation' OR LICI OR 'intracortical facilitation' OR ICF OR 'resting motor threshold' OR RMT OR 'cortical silent period' OR CSP)

**B) PRISMA flowchart of selection**

**
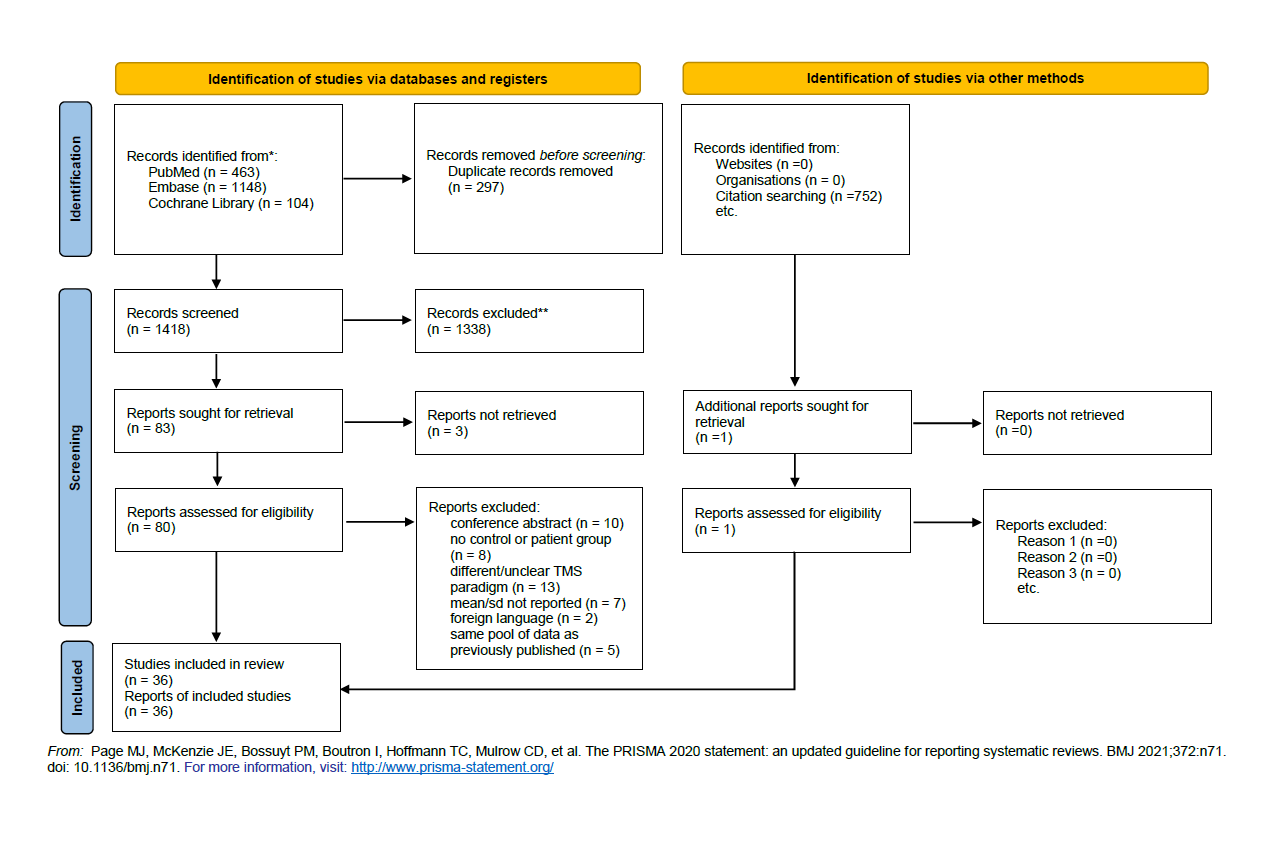
**

**Supplementary Figure 1.**

*PRISMA flowchart of selection*

**C) ICF, LICI, RMT descriptive statistics**

|  | **N studies** | **N total**  **SCH/HC** | **Mean age SCH (min-max)** | **Mean age HC**  **(min-max)** | **Mean illness duration SCH  (min-max)** | **Mean PANSS total SCH**  **(min-max)** | **Mean CPZ equivalent dose**  **(min-max)** |
| --- | --- | --- | --- | --- | --- | --- | --- |
| **ICF** | 20 | 532/498 | 30.4 (25.3-36.8) | 29.7 (20.9-37.3) | 3.64 (0-11.1) | 72.4  (50.5-92.4) | 427.5 (284.0-860.8) |
| **LICI** | 4 | 171/201 | 32.6 (30.7-33-8) | 31.9 (30.7-33.2) | 11.15  (11.1-11.2) | 68.3  (50.5-86.1) | 393.5  (373.7-413.4) |
| **RMT** | 33 | 902/932 | 32.8  (27.4-39.1) | 32.4  (20.9-42.2) | 7.5  (0-18.3) | 69.1  (50.5-101) | 420.5  (266.3-860.8) |

**Supplementary Table 1.**

*Descriptive statistics of the articles including Intracortical Facilitation (ICF), Long-Interval Intracortical Inhibition (LICI), and Resting Motor Threshold (RMT). Abbreviations: SCH = Schizophrenia, HC = Healthy Controls, CPZ equivalent dose = chlorpromazine equivalent daily antipsychotic medication dose*

**D) SICI additional results**

**D.1. Exploratory analysis: antipsychotic medication dose**

Exploratory analyses revealed a tendency-like association between SICI and medication status as well as SICI and CPZ equivalent antipsychotic dose (see Figure 2./D in the manuscript). During correlation analysis CPZ equivalent antipsychotic dose was set to 0 for all unmedicated patients. To better understand the effect of antipsychotic dose and to further differentiate between medicated and unmedicated patients two additional correlation analyses were conducted. First, we conducted a correlation between mean SICI and mean CPZ equivalent antipsychotic dose for all articles where SICI and medication dose was available (Supplementary Figure 2.). Here the trend-level association is maintained. These results however are limited since medication data was available for those articles where the patients sample consisted of medicated and unmedicated patients, but SICI was not reported separately for the two patient group. Six from the 15 plotted articles for Supplementary Figure 2. reported mean SICI for medicated an unmedicated patients together. From these six articles the mean unmedicated patient ratio was 17.39% (min =15%, max=61%), which could have a meaningful impact on the interpretation of the results.

To account for this confounding effect a second correlation analysis was conducted with those articles where mean SICI was reported separately for medicated patients and data on antipsychotic dose was available. Supplementary Figure 3. shows that even the trend level association diminishes.


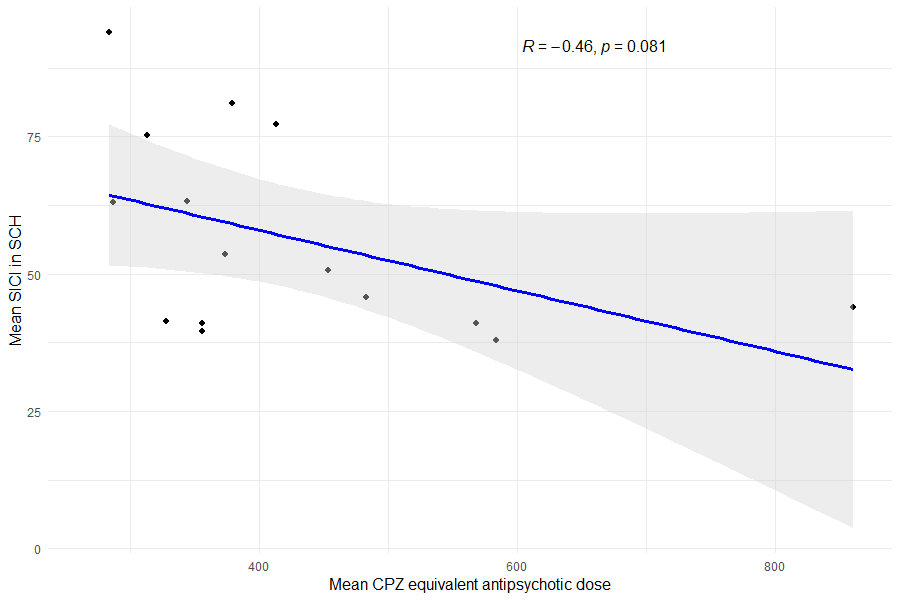


**Supplementary Figure 2.**

*Correlation between mean SICI and CPZ equivalent medication dose including data from articles where medication data was available but SICI was reported together for medicated and unmedicated patients.*

*
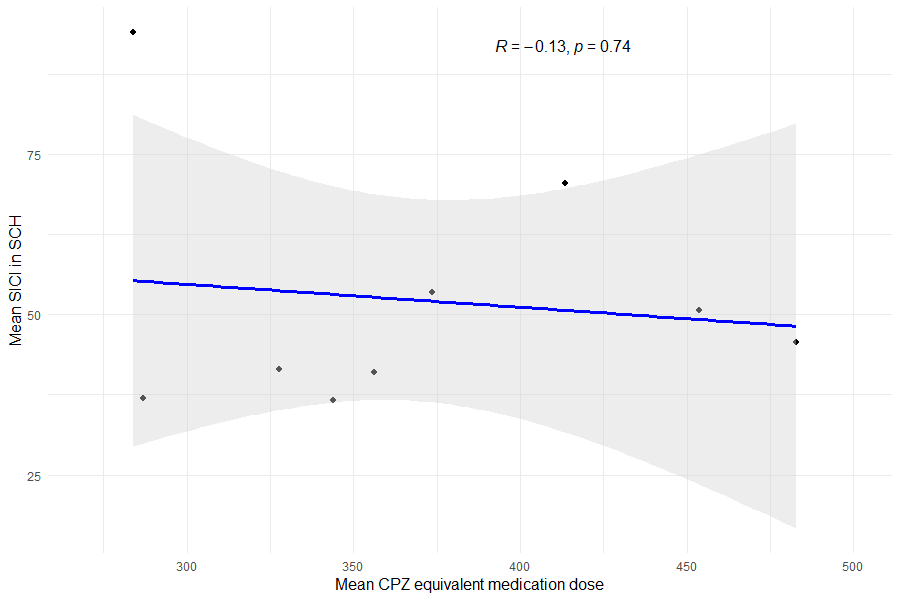
*

**Supplementary Figure 3.**

*Correlation between antipsychotic dose and mean SICI for medicated patients separately.*

**D.2. Exploratory analysis: PANSS, age, illness duration and sex**

**
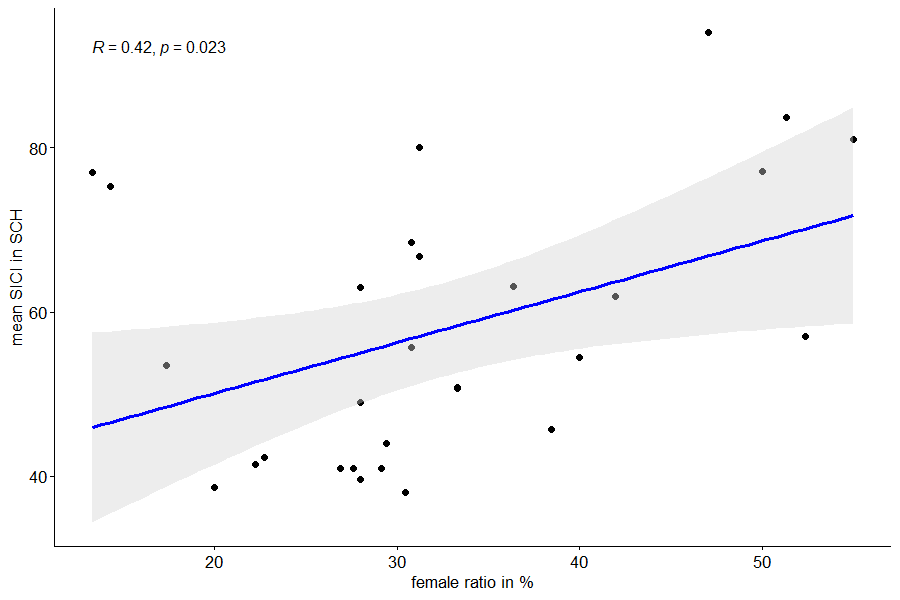

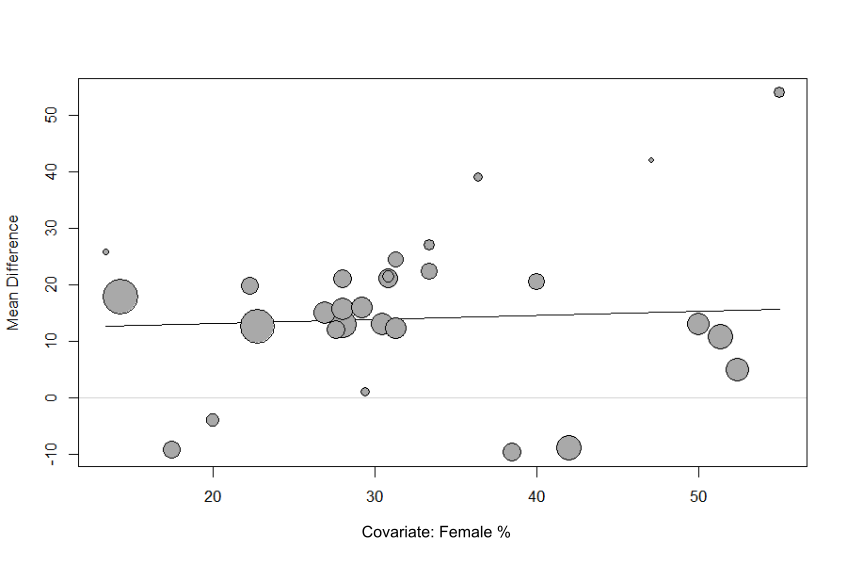
**

**Supplementary Figure 4.**

*Left: correlation between SICI and the ratio of females in the sample. Right: Result of the meta-regression model where the ratio of females was introduced as a covariate.*

*
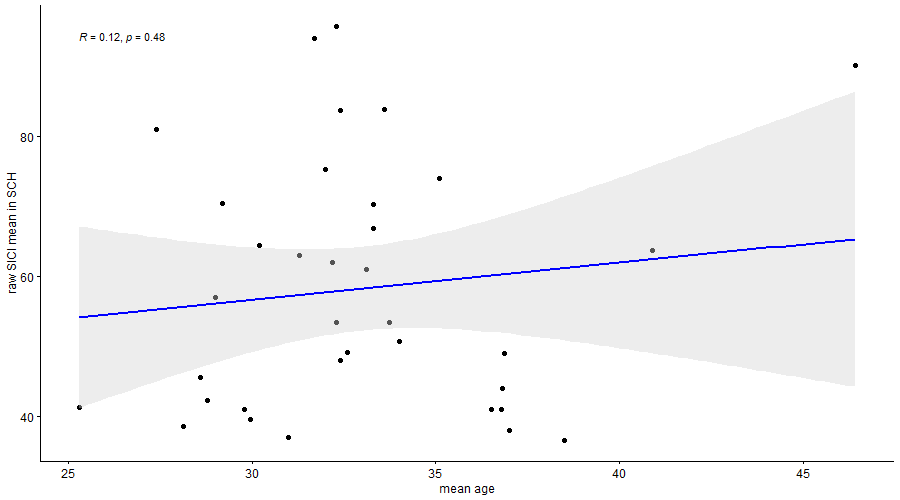
*

**Supplementary Figure 5.**

*Correlation between SICI and mean age of the patients.*

*
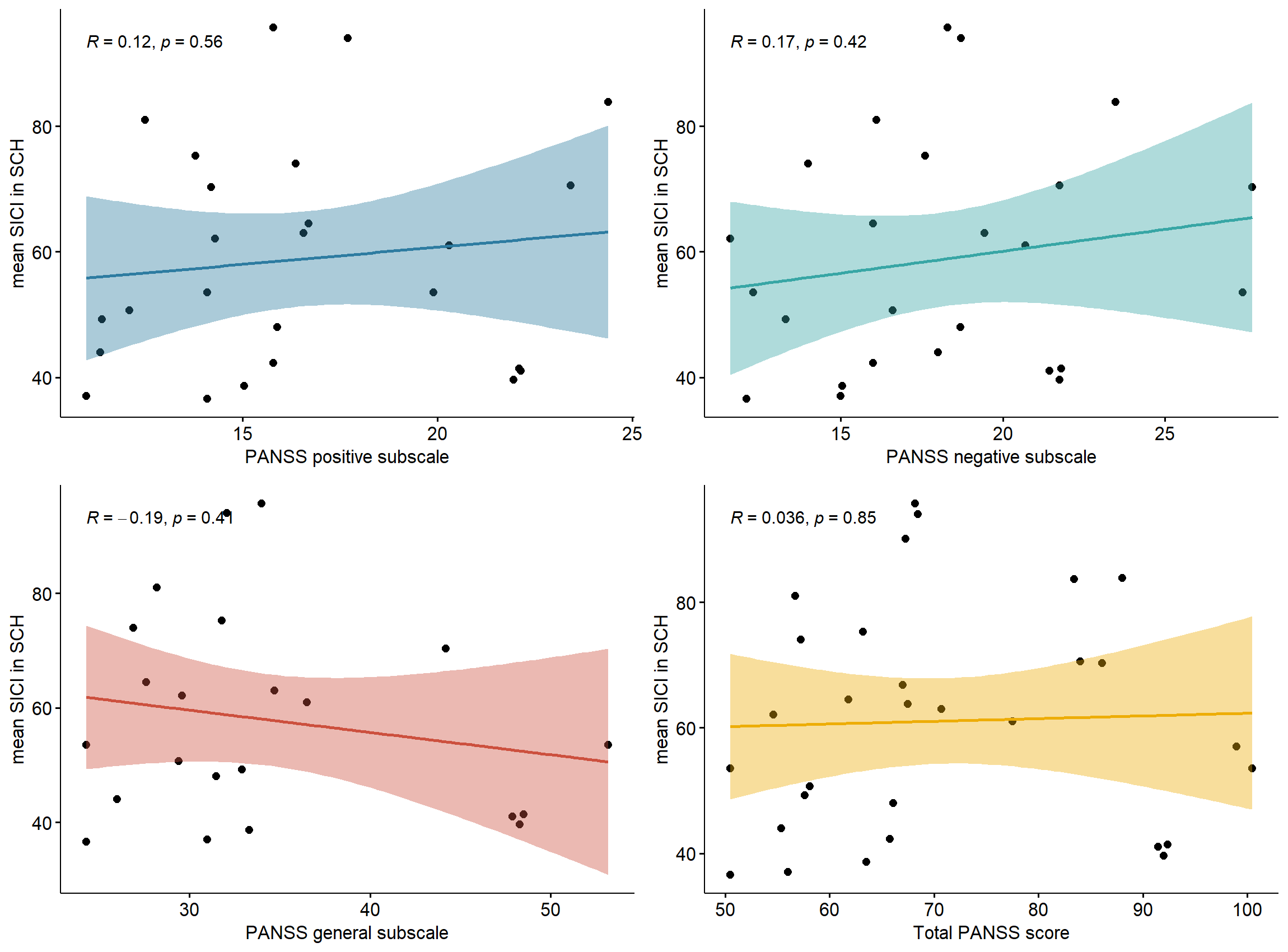
*

**Supplementary Figure 6.**

*Correlation between SICI and total PANSS scores.*

**D.3. Meta-regressions**

| **Variable** | **Estimate** | **Standard Error** | **t** | **df** | **p** |
| --- | --- | --- | --- | --- | --- |
| **Medication Dose** | -0.0078 | 0.0229 | -0.3426 | 14 | 0.7370 |
| **PANSS total** | -0.2353 | 0.1721 | -1.3670 | 21 | 0.1861 |
| **PANSS positive** | 0.0635 | 0.3170 | 0.2003 | 17 | 0.8437 |
| **PANSS negative** | -0.0254 | 0.3278 | -0.0774 | 17 | 0.9392 |
| **PANSS general** | -0.2239 | 0.3720 | -0.6021 | 15 | 0.5561 |
| **Illness duration** | -0.1379 | 0.6047 | -0.2281 | 17 | 0.8223 |
| **Age SCH** | 0.4296 | 0.7008 | 0.6130 | 27 | 0.5450 |
| **Age HC** | -0.1271 | 0.4924 | -0.2581 | 27 | 0.7983 |
| **Female %** | 0.0727 | 0.2160 | 0.3367 | 27 | 0.7389 |

**Supplementary Table 2.**

*Results of meta-regressions that we used to investigate patterns in heterogeneity for SICI. All variables were introduced to a simple meta-regression model where the dependent variable was the basic random effect meta-analytic model for SICI with mean difference as the pooled effect size.*


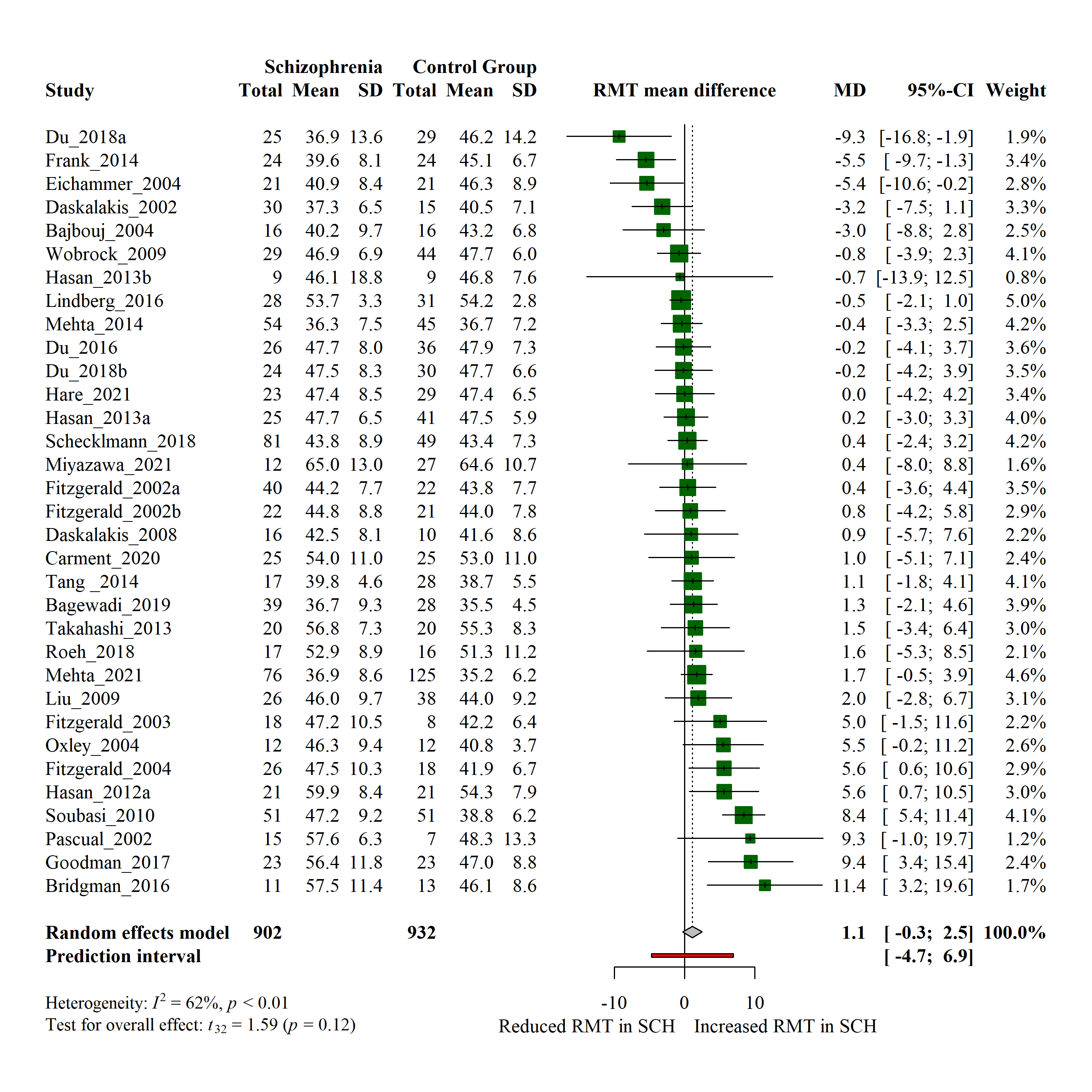
**E) RMT results**

**Supplementary Figure 7.**

*Forest plots for Resting Motor Threshold (RMT). Articles from zero to left show smaller RMT in SCH whereas articles from 0 to right show greater RMT in SCH. There is no significant difference between the two groups.*

**F) Funnel Plots**

**
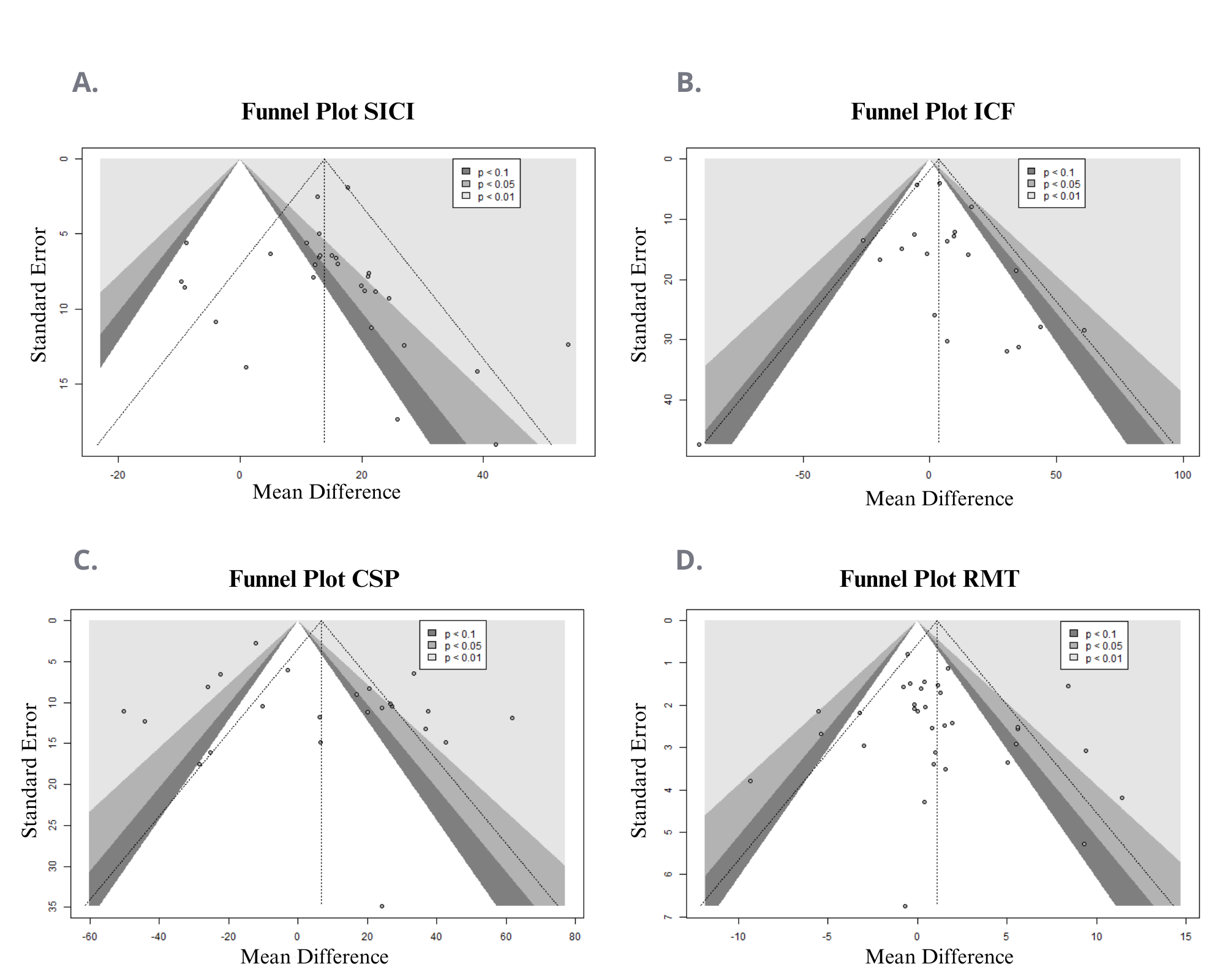
**

**Supplementary Figure 8.**

*Contour enhanced funnel plots showing no clear indication for publication bias in the case of Short-Interval Intracortical Inhibition, SICI (A), Intracortical Facilitation, ICF (B), CSP (C), and Resting Motor Threshold, RMT (D). Due to the limited number of studies available (N=4) we could not assess publication bias for Long-Interval Intracortical Inhibition, LICI.*

**G) Egger’s test**

|  | **Intercept** | **df** | **t** | **p** |
| --- | --- | --- | --- | --- |
| **SICI** | 13.88 | 27 | 0.11 | 0.91 |
| **CSP** | -14.53 | 21 | 1.73 | 0.097 |
| **ICF** | -1.06 | 18 | 0.88 | 0.39 |
| **RMT** | -0.29 | 31 | 0.92 | 0.36 |

**Supplementary Table 3.**

*Results of the Egger’s test that were used to test publication bias. Based on the results we were not able to detect significant publication bias for SICI, CSP, ICF or RMT. We were unable to complete the Egger’s test for LICI due to the limited number of studies (N=4).*
